# Minimally invasive treatment for critical multiple trauma with traumatic diaphragmatic hernia combined with pelvic and acetabular fractures: a rare case report

**DOI:** 10.3389/fmed.2026.1722340

**Published:** 2026-03-03

**Authors:** Meng-Fei Zhou, Zeng-Ying Xing, Ri-Jin Xing, Tian-Yang Li, Bi-Gang Li, Wei Liu, Yun-Yun Wu, Jun Xiong

**Affiliations:** Hainan General Hospital, Hainan Affiliated Hospital of Hainan Medical University, Haikou, China

**Keywords:** combined pelvic and acetabular fractures, HoloSight Orthopedic Surgical Robot, multidisciplinary joint surgery, severe polytrauma, traumatic diaphragmatic hernia

## Abstract

Polytrauma represents a critical emergency condition characterized by complex treatment challenges and multiple complications, constituting one of the primary causes of mortality from accidental injuries. Severe polytrauma typically results from high-energy impacts, with traffic accidents, falls from height, and other fall-related injuries being the predominant causes. Without prompt and appropriate intervention, mortality rates range from 20 to 70%. The combination of polytrauma, traumatic diaphragmatic hernia, and pelvic-acetabular fractures complicates clinical management, prolongs treatment duration, and leads to significantly elevated mortality and disability rates. This case underscores the critical importance of integrating standardized clinical trauma treatment protocols with robot-assisted minimally invasive surgery to enhance patient survival rates and promote early functional recovery.

## 1 Introduction

Polytrauma with concurrent pelvic and acetabular fractures resulting from high-energy trauma presents complex clinical challenges and significant treatment difficulties. This relatively uncommon but demanding injury pattern comprises 5.1–16.1% of all pelvic and acetabular injuries (1–10). Systematic management of these injuries is essential for optimizing postoperative functional outcomes, reducing mortality rates, and minimizing complications. Following trauma care protocol assessment guidelines, clinicians must simultaneously conduct diagnostic evaluation, implement treatment, and assess preoperative complications from soft tissue injuries surrounding the fractures, including respiratory failure, cardiac dysfunction, and hemorrhagic shock (11). The management of these complications may necessitate postponing surgical intervention for pelvic and acetabular fractures or modifying surgical approaches. This often requires collaboration with surgical subspecialties or activation of a multidisciplinary team (MDT) for preoperative assessment and planning to optimize surgical outcomes (12–20) and maximize the success rate of pelvic and acetabular fracture surgery (21). While technological advances have diversified surgical options for pelvic and acetabular fractures, traditional open reduction and internal fixation (ORIF) presents significant risks for polytrauma patients with traumatic diaphragmatic hernia (TDH) and combined pelvic-acetabular fractures. After comprehensive evaluation, our trauma and orthopedics team selected the pelvic unlocking closed reduction technique (UCRT) combined with the HoloSight Orthopedic Surgical Robot for treating these combined fractures. This approach, widely utilized in minimally invasive management of unstable pelvic fractures, minimizes operative trauma and facilitates early postoperative rehabilitation.

## 2 Materials and methods

### 2.1 General information

Patient Li, male, 30 years old, presented to the Emergency Department of Hainan Hospital Affiliated to Hainan Medical University on March 19, 2023, with “multiple traumatic pains throughout the body and limited mobility for 2 hours”. The patient sustained injuries when accidentally crushed by a truck during repair work. He contacted emergency services (120) and was transported to the hospital’s Emergency Department. The patient reported pain in the chest, abdomen, and hip regions, with movement limitations and generalized chills. Preliminary Physical Examination: Initial vital signs showed Temperature (T): 36.5 °C, Pulse (P): 115 beats per minute, Respiration (R): 14 breaths per minute, Blood Pressure (BP): 79/48 mmHg. Clinical examination revealed altered mental status, pallor, left thoracic cavity drainage tube *in situ*, decreased bilateral respiratory excursion, chest wall tenderness with multiple sites of crepitus, left waist contusion with positive local tenderness and percussion pain, significant right hip tenderness, positive pelvic compression sign, limited right lower extremity movement, decreased sensation, impaired mobility, and coldness throughout the right lower extremity; dorsalis pedis and posterior tibial arterial pulses were non-palpable.

### 2.2 Case presentation

**Figure 1:**
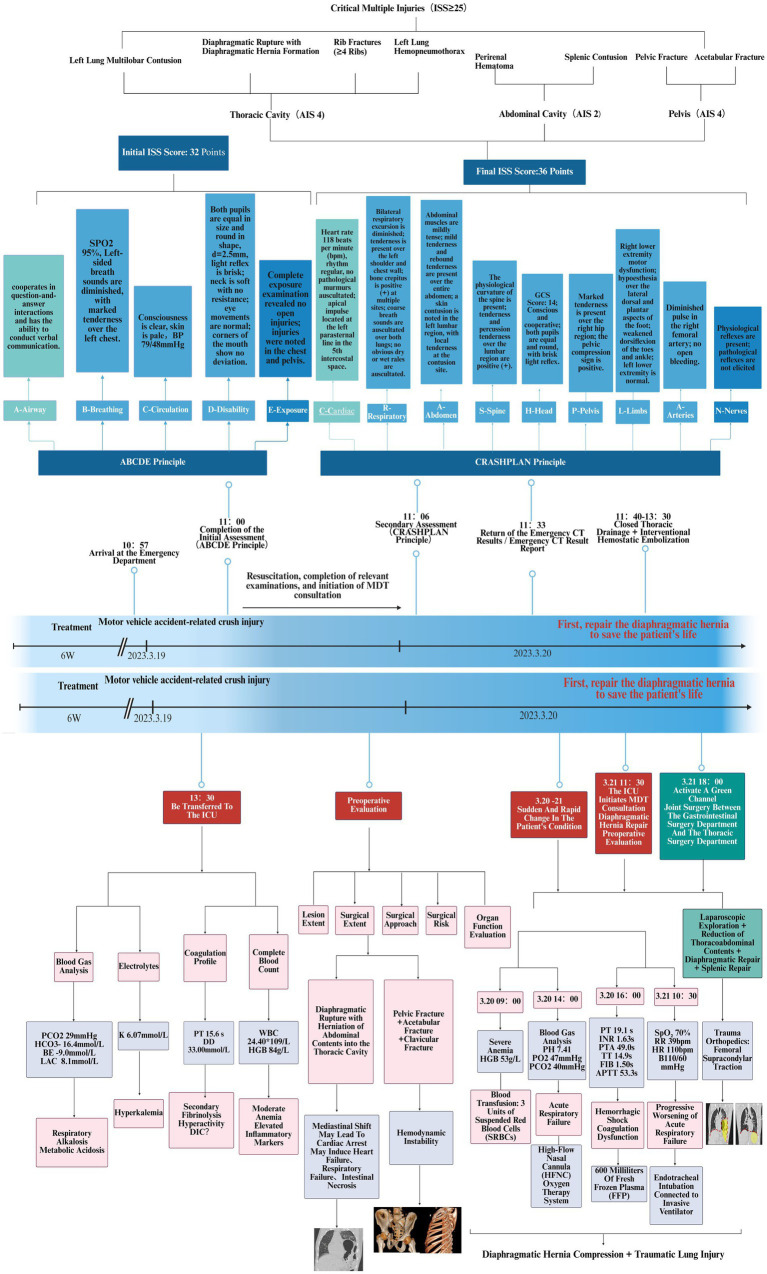
Trauma care process score and treatment flow chart for traumatic diaphragmatic hernia.

**Figure 2:**
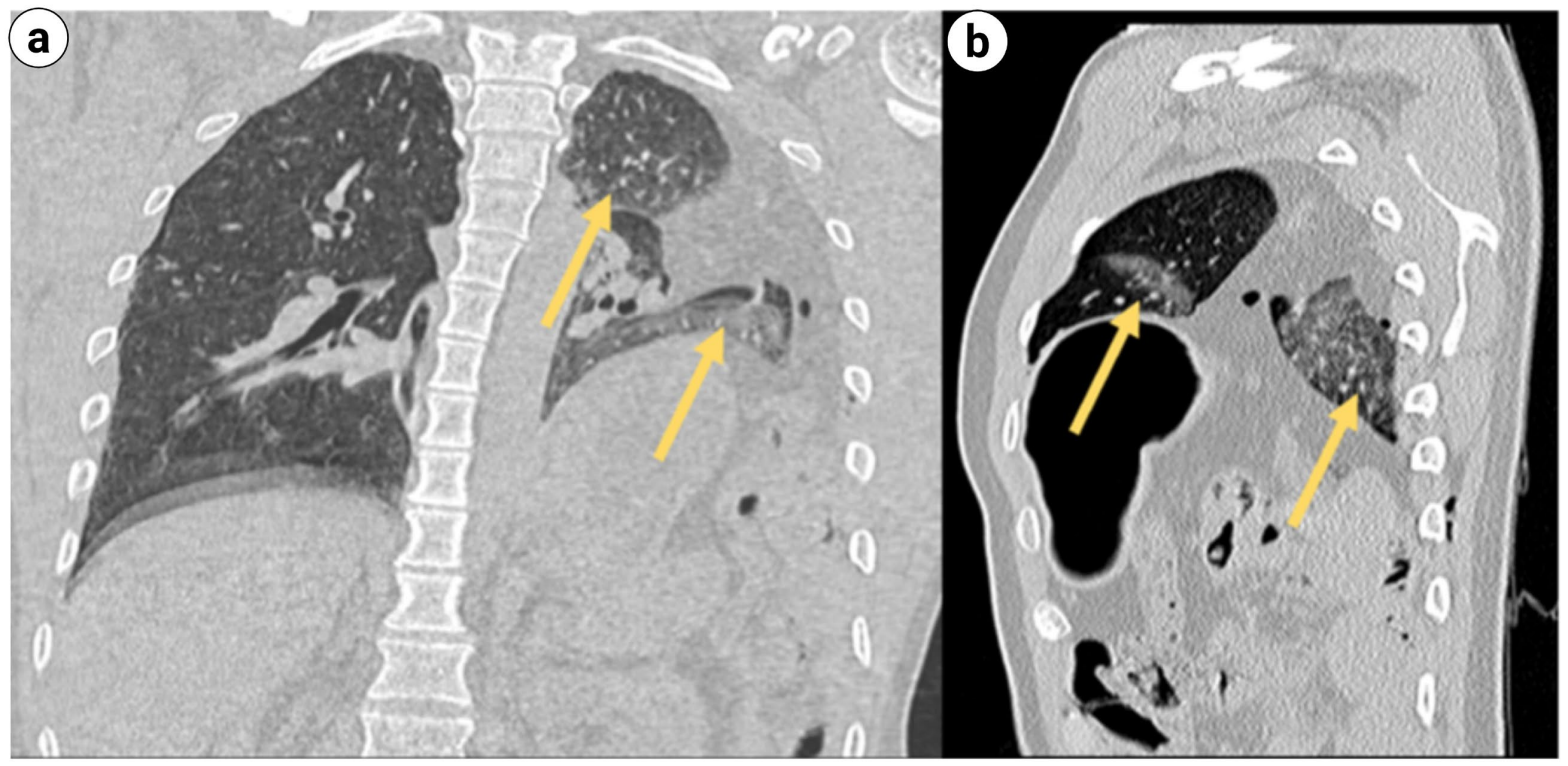
Multilobar contusion of the left lung (indicated by the yellow arrow): **(a)** chest CT coronal plane; **(b)** chest CT sagittal plane.

**Figure 3:**
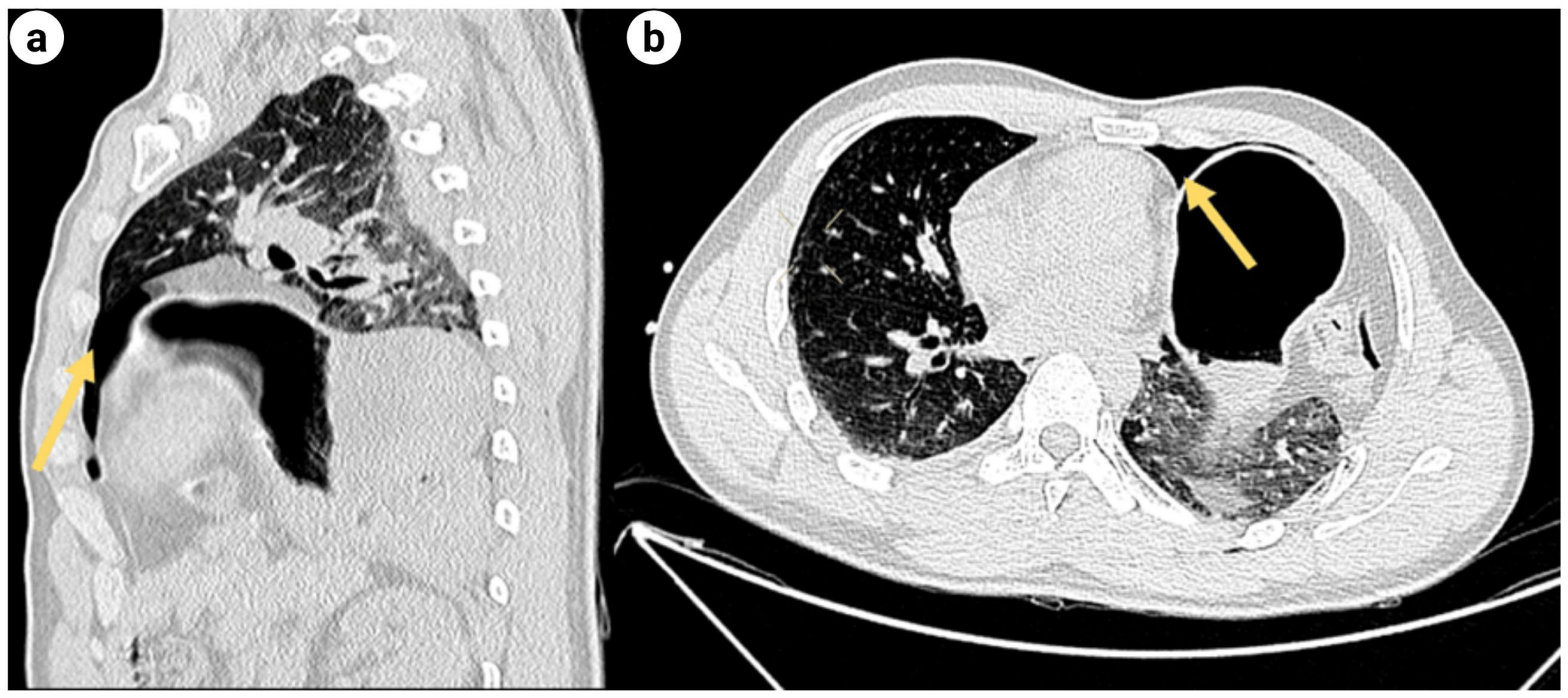
**(a)** Sagittal and axial CT scans of the chest: Left pneumothorax (denoted by the yellow arrow); **(b)** the axial CT scan of the chest: Left pleural effusion (indicated by the yellow arrow).

**Figure 4:**
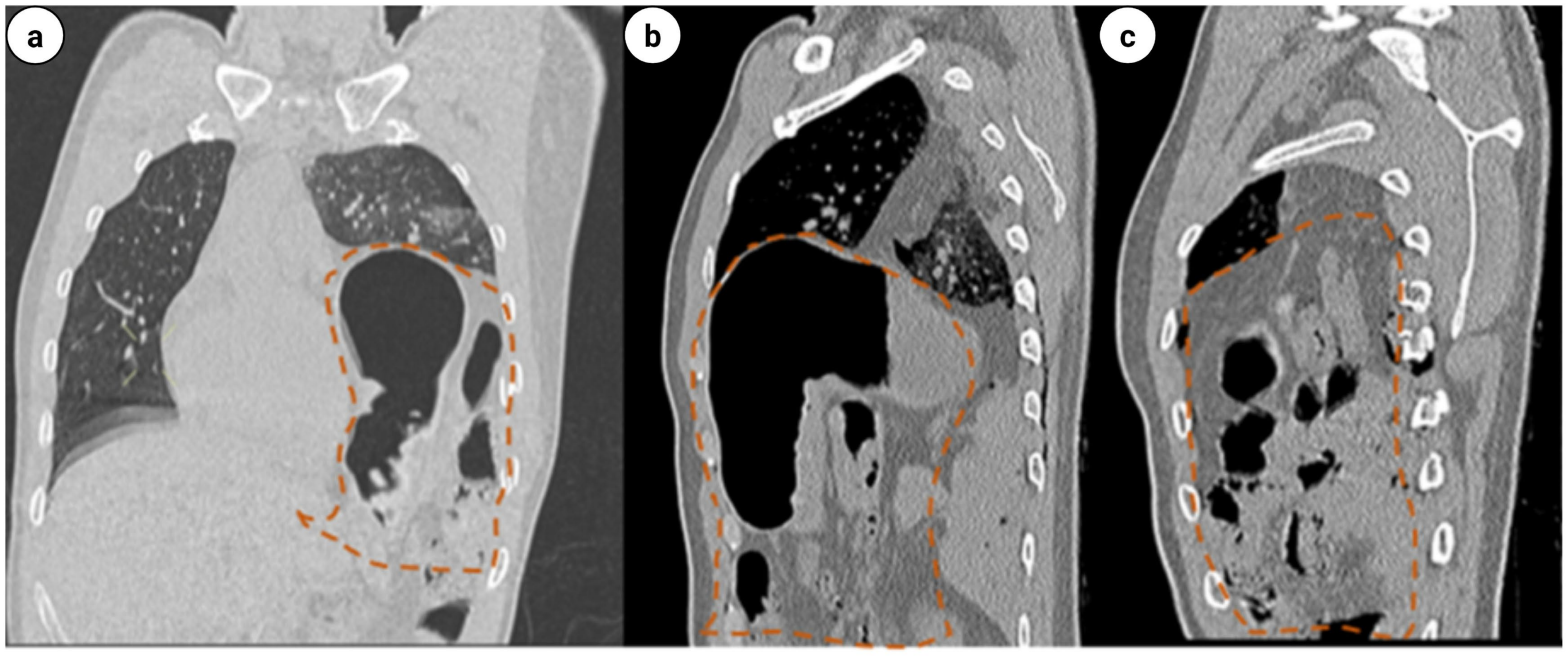
**(a)** Coronal and sagittal CT scans of the chest: left diaphragmatic rupture complicated with left diaphragmatic hernia. **(b,c)** The gastric fundus, gastric body segment, portion of small intestine, and transverse colon were identified herniating into the left thoracic cavity (within the red dashed line).

**Figure 5:**
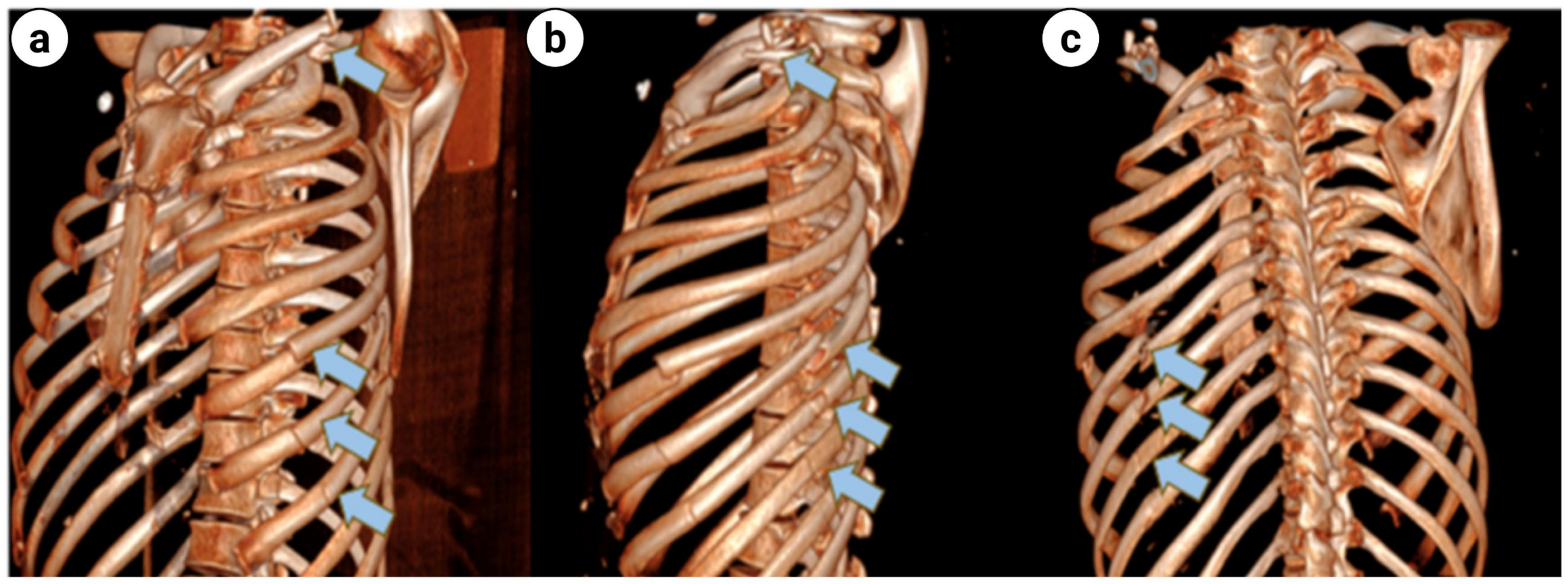
**(a)** Coronal view of chest 3D CT shows left clavicle fracture and multiple fractures of multiple left ribs; **(b)** Sagittal view of chest 3D CT shows left clavicle fracture and multiple fractures of multiple left ribs; **(c)** Dorsal view of chest 3D CT shows multiple fractures of multiple left ribs (indicated by the blue arrows).

**Figure 6:**
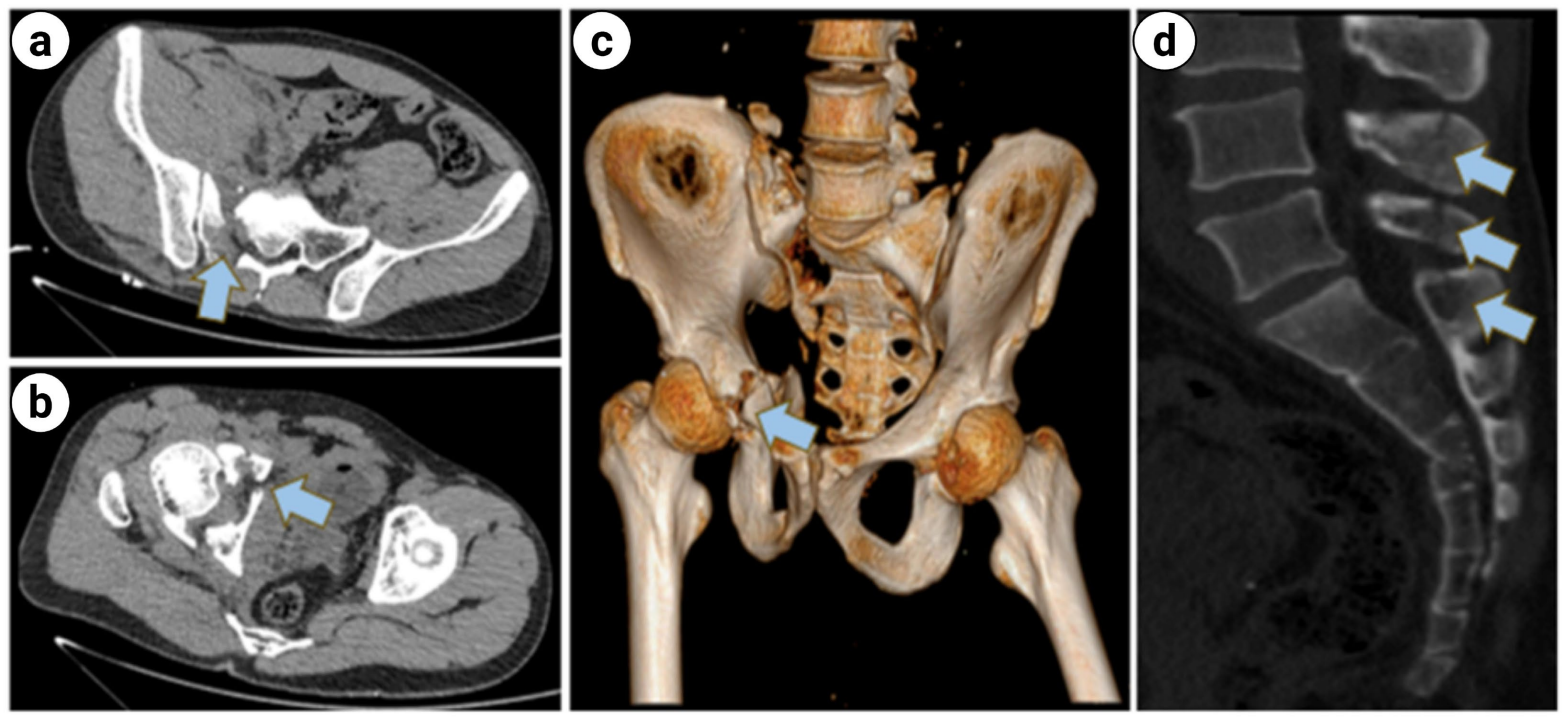
**(a,b)** Axial CT scan of the pelvis: Fractures of the right sacral ala; **(c)** 3D CT scan of the pelvis: fracture of the acetabulum and superior pubic ramus; **(d)** sagittal CT scan of the sacrococcygeal region: The sagittal CT scan of the sacrococcygeal region demonstrates partial fractures of the lumbar spine accessories (indicated by the blue arrows).

**Figure 7:**
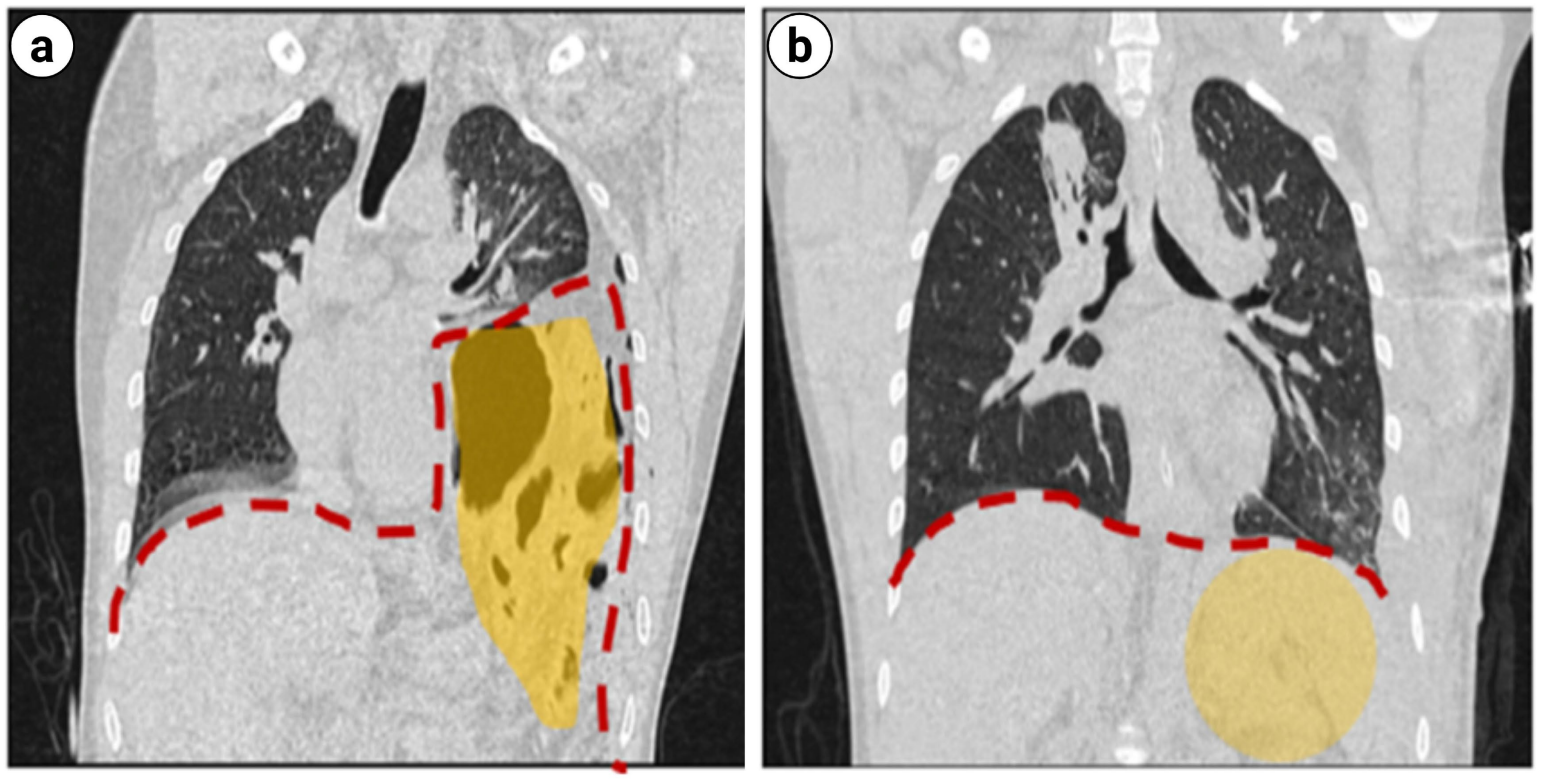
Coronal CT scan of the chest: **(a)** Preoperative traumatic diaphragmatic hernia reduction. **(b)** Postoperative traumatic diaphragmatic hernia reduction (within the yellow shaded area).

**Figure 8:**
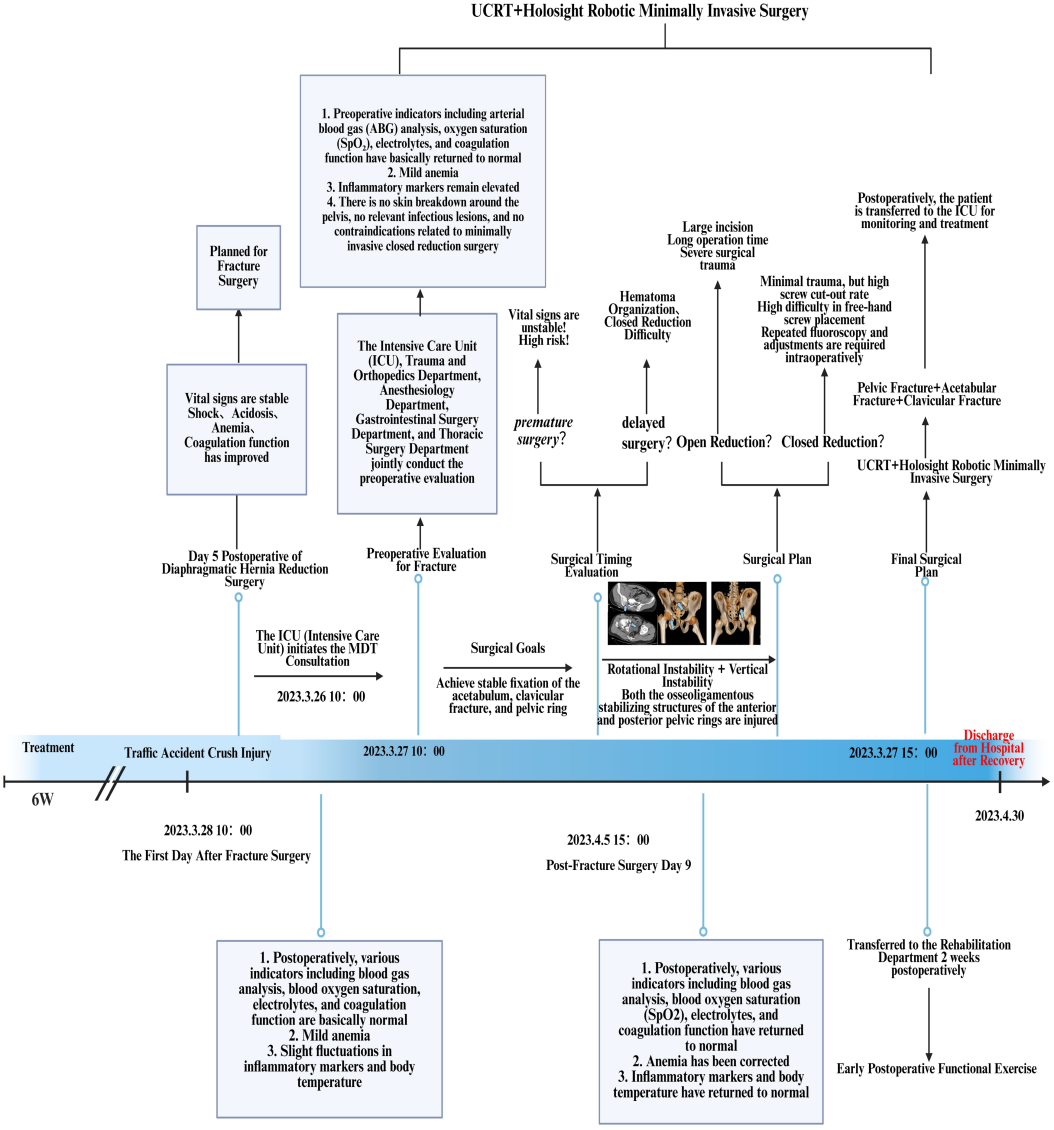
Treatment flow chart for combined pelvic and acetabular fractures.

**Figure 9:**
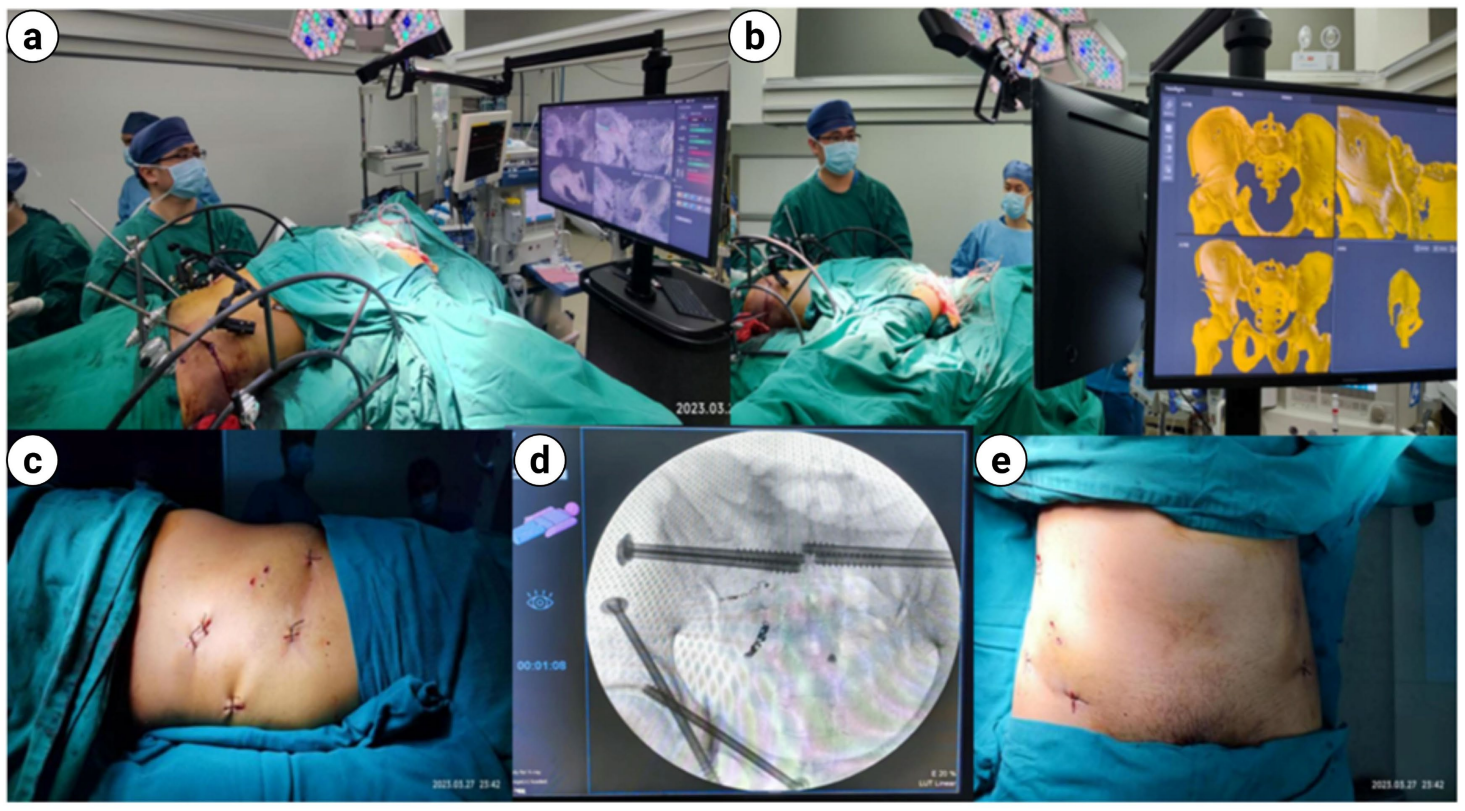
**(a,b)** The interface for performing pelvic reduction and screw placement using intraoperative UCRT combined with HoloSight trauma orthopedic surgical robots; **(c–e)** intraoperative fluoroscopic imaging following minimally invasive incision and screw placement under navigation of the HoloSight trauma orthopedic surgical robot in UCRT.

**Figure 10:**
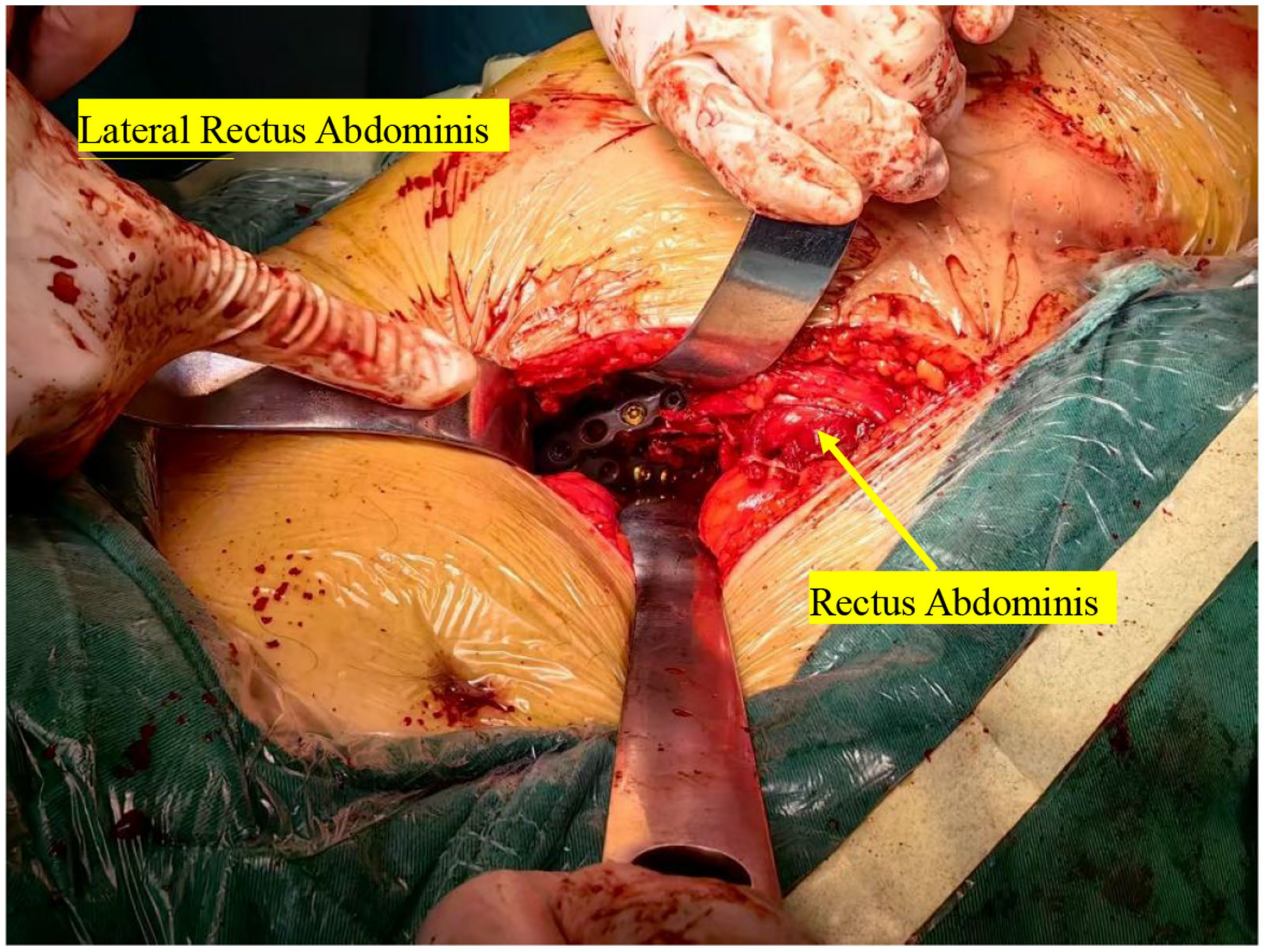
Intraoperative images of traditional ORIF (as indicated by the yellow arrow).

**Figure 11:**
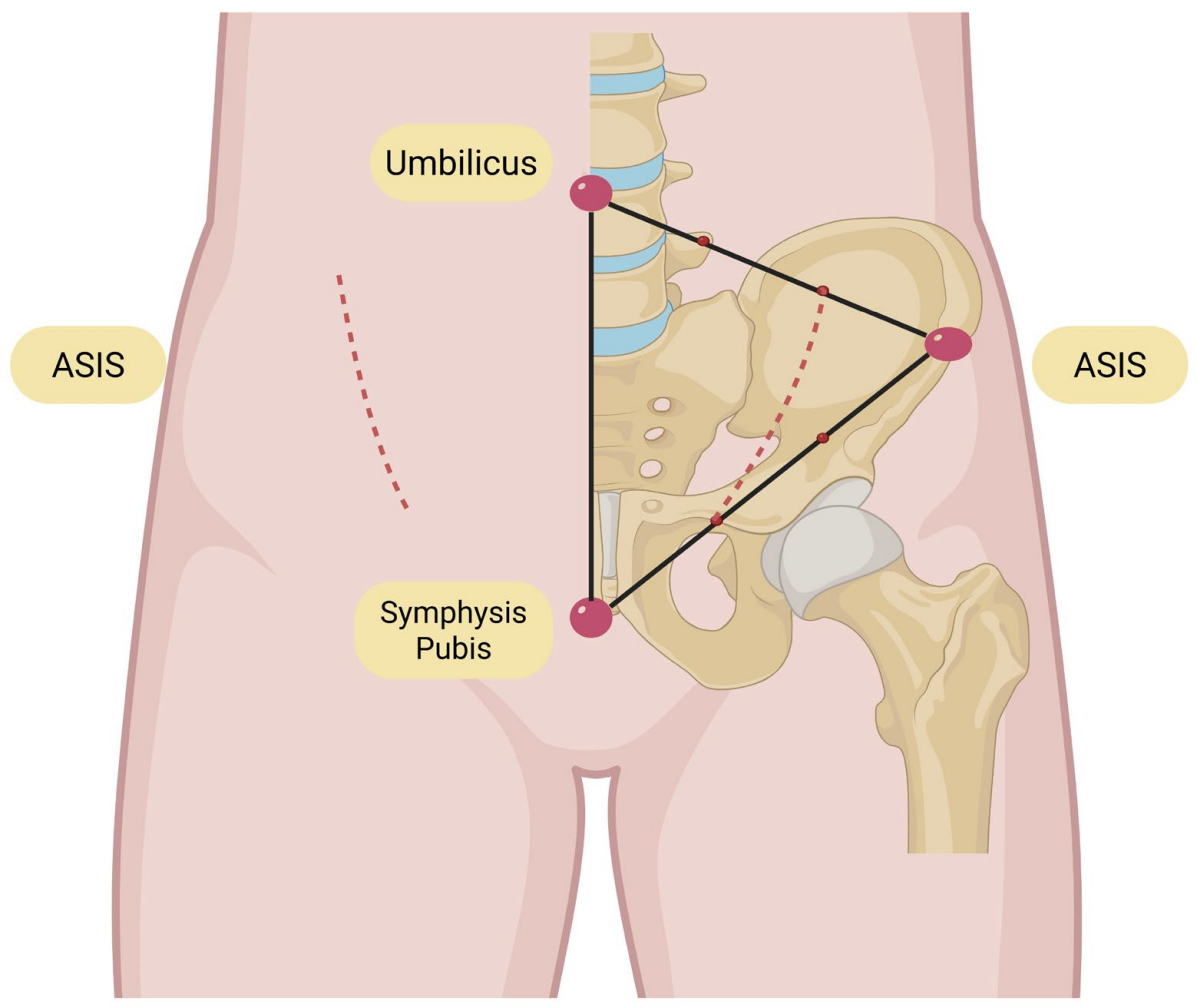
Illustration of the lateral approach through the abdominal rectus muscle.

**Figure 12:**
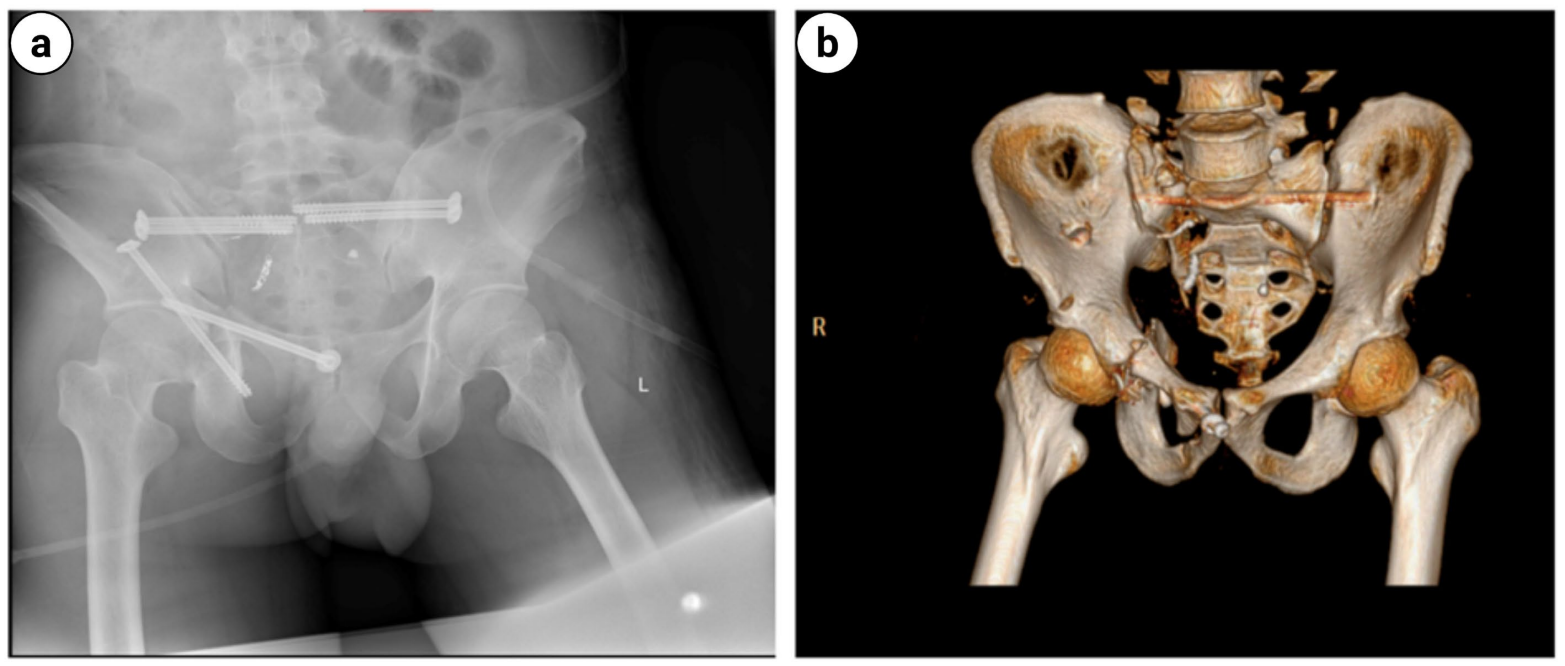
Postoperative day-2 follow-up pelvic radiograph and three-dimensional computed tomography (3D CT) revealed excellent quality of fracture reduction with appropriately positioned screws: **(a)** Pelvic radiograph; **(b)** Three-dimensional pelvic computed tomography.

Upon admission to the Emergency Department (ED), the hospital promptly activated the severe trauma management protocol. Following Advanced Trauma Life Support (ATLS) principles, the emergency physician completed the primary assessment within 5 min according to the ABCDE principle (Figure 1). The initial Injury Severity Score (ISS) was 32. Given the suspected chest and pelvic injuries complicated by hemorrhagic shock, initiate advanced life support measures, including continuous cardiac monitoring, oxygenation support, establishment of intravenous access, intravenous infusion of sodium lactate Ringer’s solution for fluid resuscitation in shock management, and continuous infusion of norepinephrine to maintain hemodynamic stability. Concurrently, blood samples are collected for comprehensive laboratory testing, encompassing complete blood count, hepatic and renal function tests, serum electrolytes, coagulation profile, and blood typing. Post-resuscitation, the patient’s systolic blood pressure increased to 91 mmHg, and diastolic pressure to 63 mmHg, indicating a partial recovery of circulatory function. Subsequently, the emergency team adhered to the *CRASHPLAN* guidelines (Figure 1), conducting a systematic secondary assessment covering cardiovascular, respiratory, abdominal, spinal, cranial, pelvic, limb, arterial, and neurological examinations (22) (Figure 1). Additionally, a whole-body contrast-enhanced computed tomography (CT) scan from the skull to the pelvis was performed to exclude potential traumatic injuries. A multidisciplinary team (MDT) consultation was promptly initiated to formulate a comprehensive treatment plan, marking the completion of the initial assessment process. Specialists from critical care medicine, gastrointestinal surgery, thoracic surgery, trauma orthopedics, and anesthesiology convened to evaluate the patient’s condition comprehensively. The emergency CT results revealed: multi-lobar contusion of the left lung (Figure 2), left pneumothorax and pleural effusion (Figure 3), left diaphragmatic hernia (Figure 4), multiple fractures of the left clavicle and ribs (Figure 5), and fractures of the right sacral alae, acetabulum, superior pubic ramus, and partial lumbar appendages (Figure 6). The Abbreviated Injury Scale-Injury Severity Score (AIS-ISS) was re-evaluated (Table 1). The final ISS was 36, classifying the case as critical polytrauma (23). During tertiary assessment, severe left lung tissue compression from pneumothorax was identified. Immediate closed thoracic drainage was performed, stabilizing vital signs within 20 min, followed by interventional embolization for hemostasis. The trauma green channel was activated, and the patient was transferred to the Intensive Care Unit (ICU). Initial ICU laboratory results (Table 2) indicated respiratory alkalosis with metabolic acidosis, type A lactic acidosis, hyperkalemia, secondary fibrinolysis, disseminated intravascular coagulation (DIC), moderate anemia, and elevated inflammatory markers. The MDT determined surgical intervention was necessary for the left TDH and combined pelvic-acetabular fractures. Left TDH posed risks of mediastinal shift potentially causing cardiac arrest, heart failure, and respiratory failure, while pelvic-acetabular fractures threatened hemodynamic stability. Early multidisciplinary surgery was deemed essential for survival. The MDT evaluated five aspects: “lesion scope – surgical scope – surgical approach – surgical risk – organ function (comorbidities)”. On day 2 in ICU, the patient experienced two episodes of acute respiratory failure with hemoglobin dropping to 53 g/L, indicating significant blood loss. Following blood transfusions and respiratory support, a second MDT consultation attributed the respiratory failure to TDH-induced lung compression combined with traumatic injury. Enhanced Recovery After Surgery (ERAS) protocols were implemented, including acute pain control, nutritional assessment, prophylactic antibiotics, bowel preparation, blood management, and anesthetic planning. The surgical teams performed laparoscopic exploration with reduction of thoracic and abdominal contents, diaphragmatic repair, and splenic repair, while trauma orthopedics performed supracondylar femoral traction. Laparoscopy revealed herniated abdominal contents causing 60% lung compression, a 10.0 cm diaphragmatic tear, and splenic lacerations. The 4-h operation had 600 mL blood loss, requiring transfusion of plasma, cryoprecipitate, and packed red cells. Post-operative chest CT confirmed successful reduction (Figure 7), with improved biochemical parameters (Table 3) showing corrected shock and acidosis, though mild anemia and inflammation persisted. By post-operative day 5, the patient stabilized sufficiently for the third MDT to approve pelvic fracture surgery. Following evidence-based guidelines for polytrauma patients (Grade 2A) (24), minimally invasive surgery was selected using the Unlocking Closed Reduction Tool (UCRT) with HoloSight surgical robot, (Figure 8) shown to improve outcomes versus traditional approaches (25). The procedure was performed on day 7 post-TDH surgery (Tables 4, 5), including ORIF of the clavicular fracture followed by robot-assisted precise reduction of pelvic-acetabular fractures (Figures 9–11). This innovative technique developed by He et al. (26, 27) addresses previous limitations in minimally invasive pelvic surgery. The 2-hour operation required minimal fluoroscopy and blood loss. Post-operative imaging confirmed anatomical reduction (Figure 12), and the patient transferred to rehabilitation after 2 weeks of recovery with improved clinical parameters.

**Table 1:** Final ISS score for polytrauma (ISS: 36).

Injury site	AIS score	ISS score: 36
Chest (AIS 4)	Multilobar contusion of left lung (AIS 4)	16
	Hemopneumothorax of left lung (AIS 3)	
	Diaphragmatic rupture with diaphragmatic hernia (AIS 4)	
	Rib fractures (>4 ribs) (AIS 3)	
Abdomen (AIS 2)	Perirenal hematoma (AIS 2)	4
	Splenic contusion (AIS 2)	
Pelvis (AIS 4)	Crush fracture of pelvis (AIS 4)	16

**Table 2:** Preoperative biochemical markers for traumatic diaphragmatic hernia repair.

Vital signs	Blood gas analysis	Electrolytes	Coagulation function tests	Complete blood count (CBC)	Others
T 37.2 °C	PH 7.36	K 6.07 mmol/L	PT 15.6s	WBC 24.40*109/L	Hs-NT 0.035ug/L
P 118tpm	PO2 120 mmHg	Ca 2.02 mmol/L	APTT 41.7s	NE% 86.4%	NT-ProBNp 12 ng/L
R 20tpm	PCO2 29 mmHg	Mg 0.67 mmol/L	DD 33.00 mmol/L	LYM% 7.0%	CRP 8.76 mg/L
BP cannot be measured	SpO2 99.1%		FIB 1.68 g/L	PLT 236*109/L	PCT 11.64 ng/mL
	HCO3–16.4 mm/L		FDP 157 μg/L	HGB 84 g/L	IL-6 201 pg./mL
	BE −9.0 mmol/L				
	LAC 8.1 mmol/L				

**Table 3:** Biochemical indicators after traumatic diaphragmatic hernia repair.

Vital signs	Blood gas analysis	Electrolytes	Coagulation function tests	Complete blood count (CBC)	Others
T 37.2 °C	PH 7.40	K 3.97 mmol/L	PT 13.4s	WBC 9.25*109/L	Hs-TNT 0.030 ug/L
P 93tpm	PO2 128 mmHg	Na 136.7 mmol/L	APTT 54.6s	NE% 86.1%	NT-ProBNp 663 ng/mL
R 18tpm	PCO2 43 mmHg	Ca 1.89 mmol/L	FIB 4.388 g/L	LYM% 7.2%	CRP 118.48 mg/L
BP 112/70 mmHg	SpO2 96%	Mg 0.94 mmol/L		PLT 57*109/L	IL-6 59.44 pg./mL
	HCO3-26.6 mm/L			HGB 95 g/L	PCT 9.230 ng/mL
	BE −1.6 mmol/L				
	LAC 1.8 mmol/L				

**Table 4:** Preoperative biochemical indicators of pelvic and acetabular fractures.

Vital signs	Blood gas analysis	Electrolytes	Coagulation function tests	Complete blood count (CBC)	Others
T 37.2 °C	PH 7.39	K 4.26 mmol/L	PT 12.4s	WBC13.84*109/L	CRP 167.81 mg/L
P 76tpm	PO2 123 mmHg	Na 129.6 mmol/L	APTT 31.4s	NE% 77.5%	IL-6 62.46 pg/mL
R 18tpm	PCO2 44 mmHg		FIB 8.47 g/L	LYM% 9.1%	PCT 0.831 ng/mL
BP 119/58 mmHg	SpO2 97%			PLT 309*109/L	
	HCO3-26.6 mm/L			HGB 106 g/L	
	BE 2.0 mmol/L				
	LAC 2.0 mmol/L				

**Table 5:** Postoperative biochemical indicators on the first day for pelvic and acetabular fractures.

Vital signs	Blood gas analysis	Electrolytes	Coagulation function tests	Complete blood count (CBC)	Others
T 38 °C	PH 7.35	K 4.80 mmol/L	PT 13.2s	WBC17.42*109/L	CRP 169.65 mg/L
P 94tpm	PO2 132 mmHg	Na 134.5 mmol/L	APTT 36.3s	NE% 87.8%	IL-6141.5 pg./mL
R 18tpm	PCO2 37 mmHg	Ca 2.12 mmol/L	FIB 7.06 g/L	LYM% 4.9%	PCT 0.664 ng/mL
BP 122/70 mmHg	SpO2 97%			PLT 309*109/L	
	HCO3-20.4 mm/L			HGB 101 g/L	
	BE −4.7 mmol/L				
	LAC 1.8 mmol/L				

## 3 Clinical outcome

**Figure 13:**
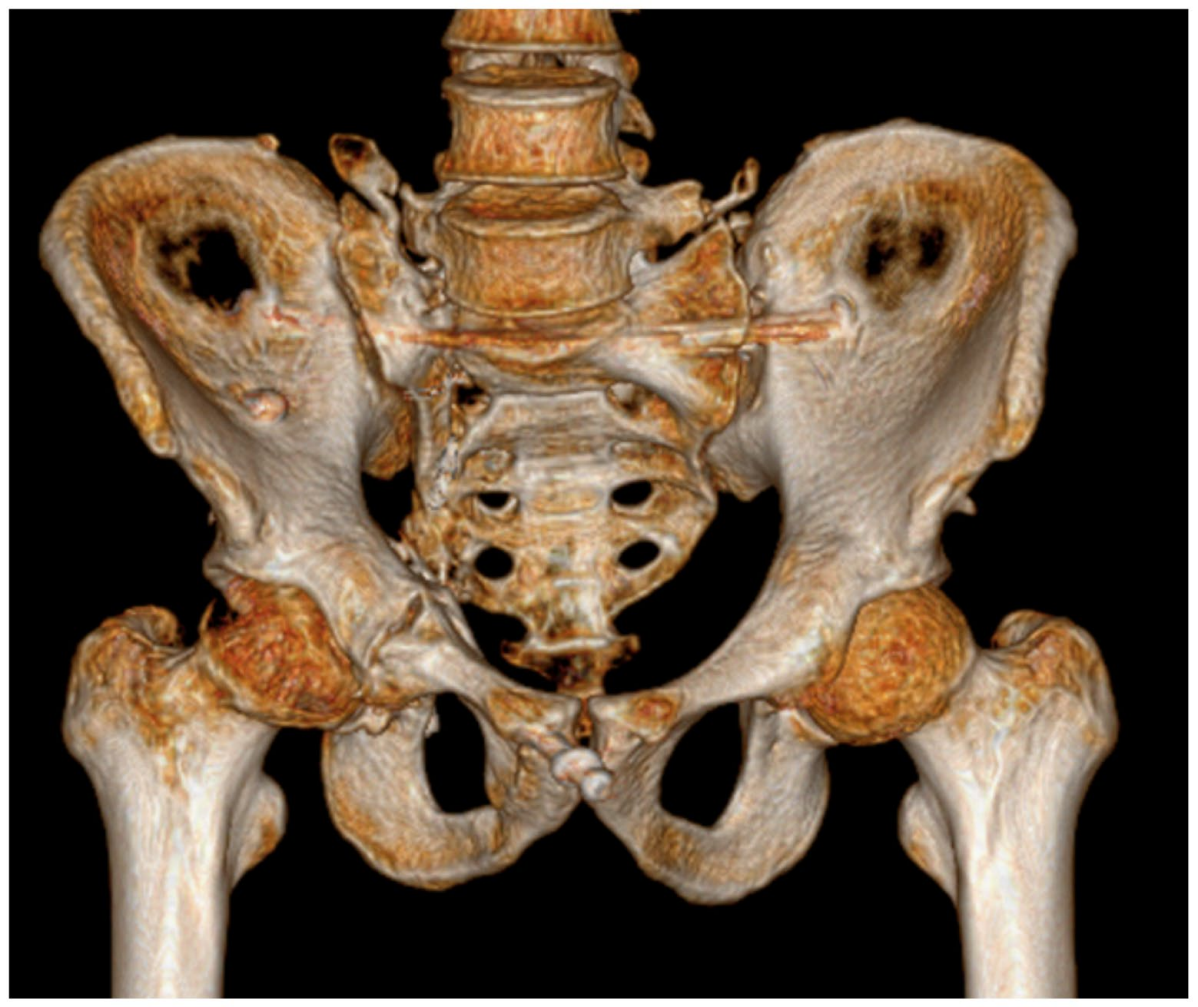
Two years postoperatively, pelvic three-dimensional computed tomography demonstrates good healing of the pelvic and acetabular fractures.

**Figure 14:**
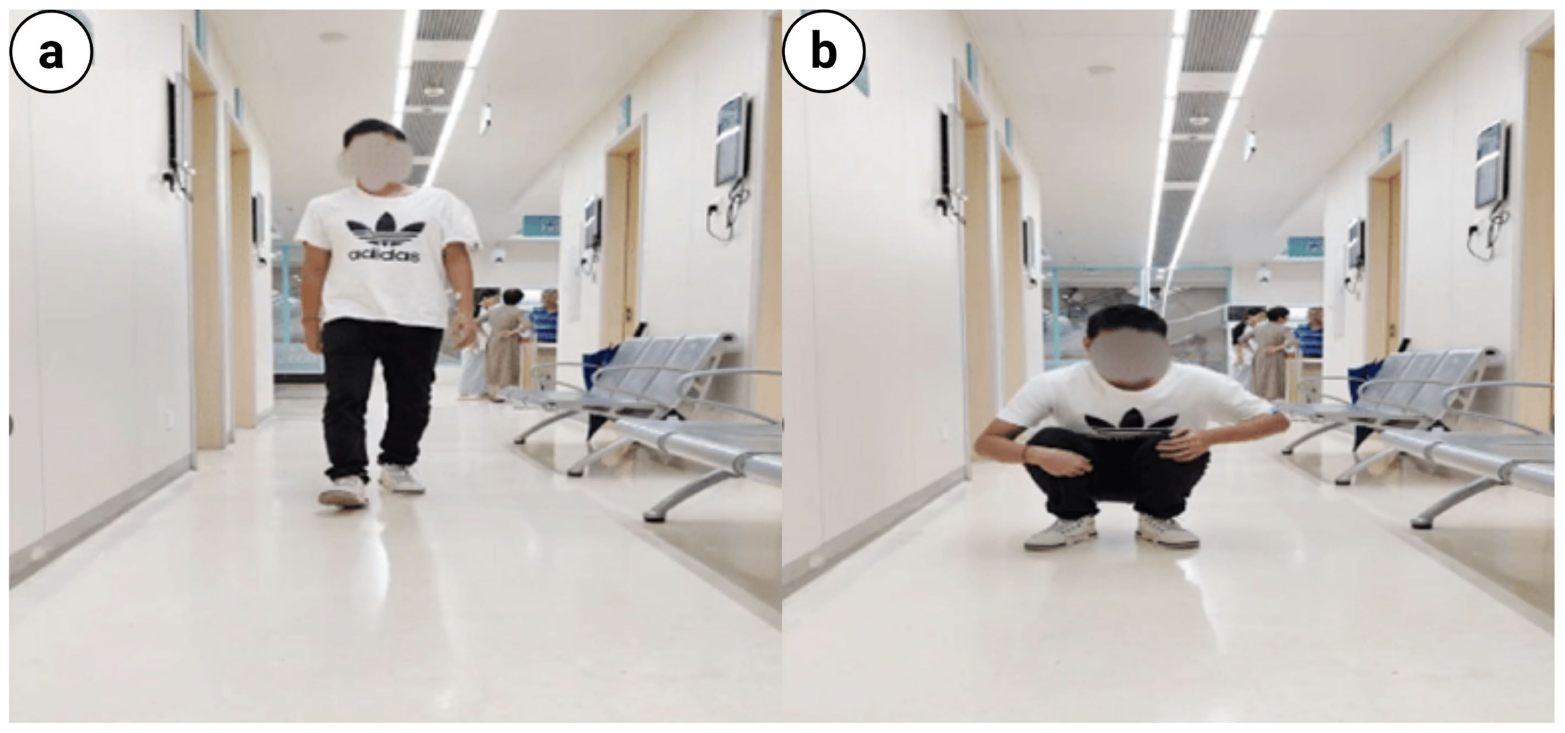
The gross functional position of the patient indicated favorable pelvic functional recovery: **(a)** Standing position; **(b)** Squatting position.

Two weeks post-surgery, the patient demonstrated improved clinical indicators and was subsequently transferred from the ICU to the rehabilitation department for acupuncture, physical therapy, and early functional rehabilitation. The patient was discharged on April 30, 2023. Following discharge, the abdominal surgical incision showed proper healing without signs of erythema or exudation; bowel sounds remained normal, a perineal examination revealed no abnormalities, and defecation patterns were regular. The pelvic, acetabular, and clavicular fractures demonstrated satisfactory healing progression. During the two-year post-discharge period, the patient attended regular follow-up appointments at two-month intervals. At the final assessment, clinical efficacy was evaluated using the Majeed scoring system, which considers pain (25 points), work capacity (20 points), sitting ability (10 points), sexual function (4 points), and standing ability (33 points; including auxiliary walking, gait, and walking distance, each scored at 11 points). The degree of clinical and neurological function improvement was assessed accordingly. Additionally, the Harris score was employed to evaluate clinical efficacy across four domains: pain, function, deformity, and joint range of motion. The scores for pelvic and hip joint functions were as follows: Majeed pelvic function score (28) was 92 points, classified as excellent (full score 100; 85–100 as excellent, 70–84 as good, 55–69 as fair, and less than 55 as poor). The Harris hip score (29) was 88 points, also classified as excellent (full score 100; 90–100 as excellent, 80–90 as good, below 80 as fair). These assessments provide a comprehensive evaluation of the patient’s functional recovery and joint performance post-treatment. Pelvic 3D CT imaging confirmed optimal screw positioning, with no evidence of loosening, and complete resolution of fracture lines (bone healing grade: Grade I) (Figure 13). No long-term complications, such as heterotopic ossification or avascular necrosis of the femoral head, were observed. The patient has regained normal ambulatory function (Figure 14) and has restored squatting and standing capabilities.

## 4 Pelvic unblocking and realignment system (UCRT) integrated with Holosight robotic-assisted minimally invasive pelvic surgery technique

**Figure 15:**
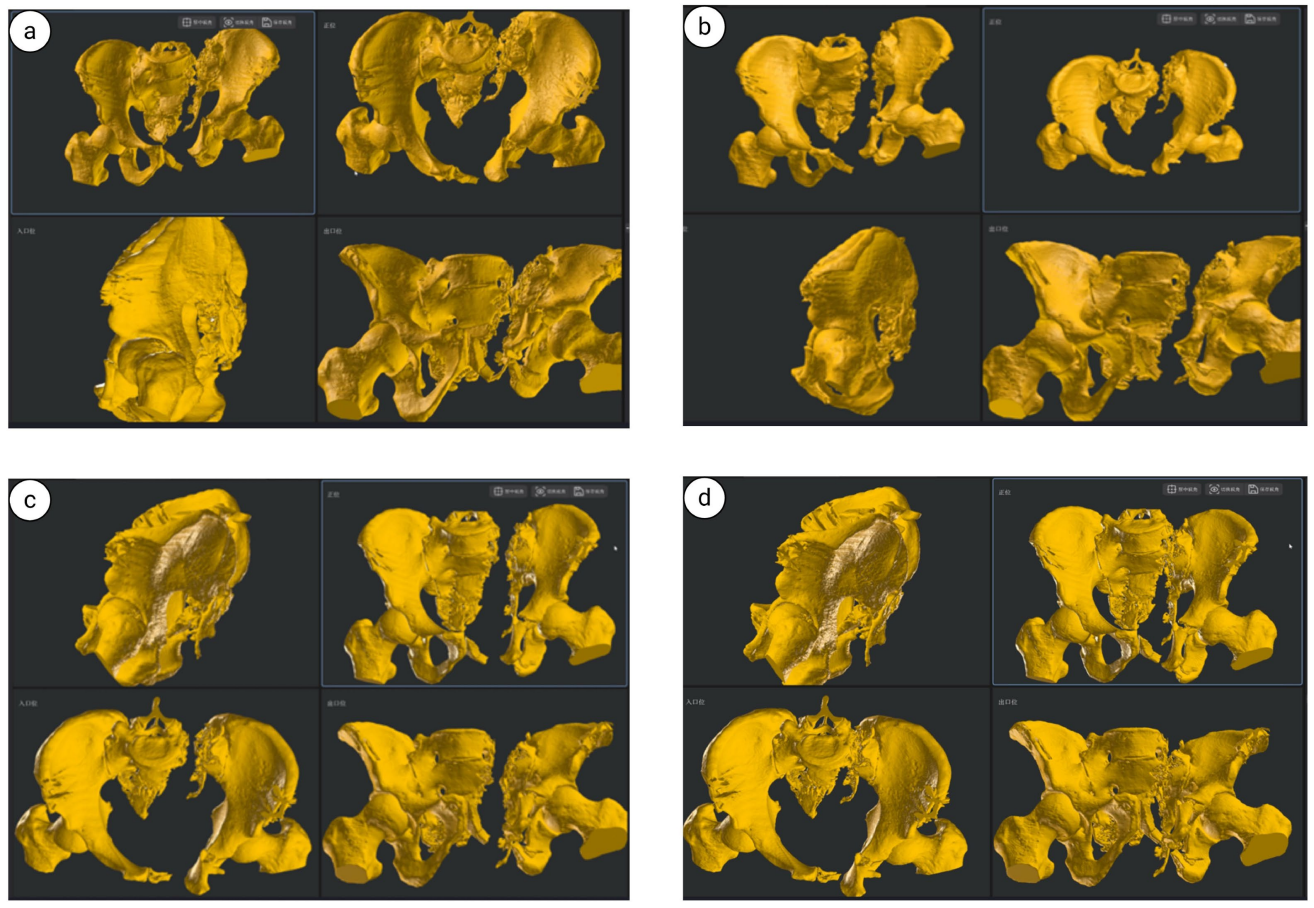
Illustration of pelvic fracture reduction using UCRT combined with HoloSight robotic system: **(a)** preoperative pelvic fracture reduction; **(b)** lateral external traction; **(c)** Loosening of the locking mechanism; **(d)** completion of closed reduction.

**Figure 16:**
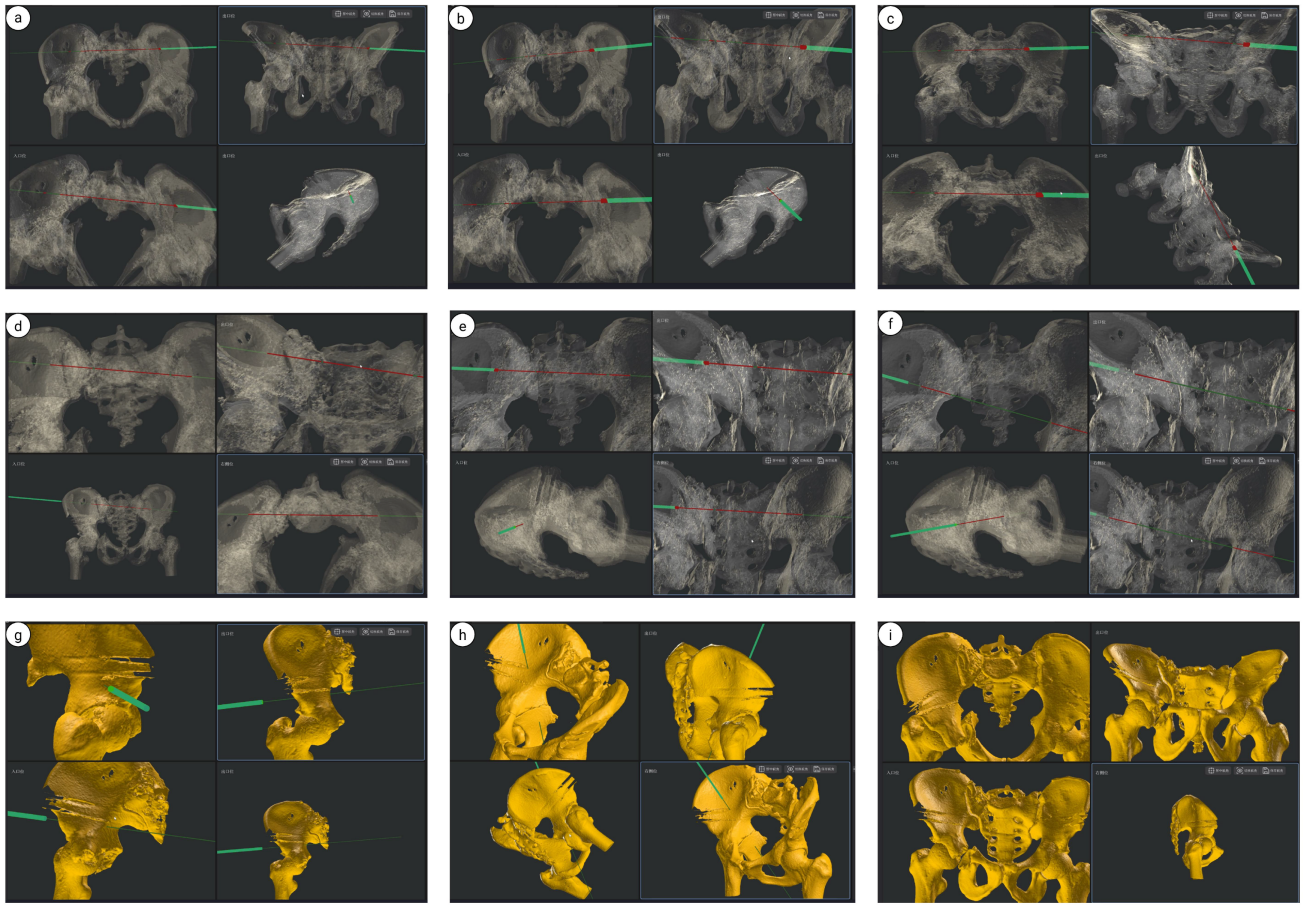
Pelvic ring fixation under HoloSight robotic navigation: **(a–f)** preoperative screw placement pathway for pelvic acetabular surgery. **(g,h)** Intraoperative screw placement pathway for acetabular fractures. **(i)** Intraoperative virtual pelvis positioning, outlet view, inlet view, and lateral view imaging for fracture reduction.

**Figure 17:**
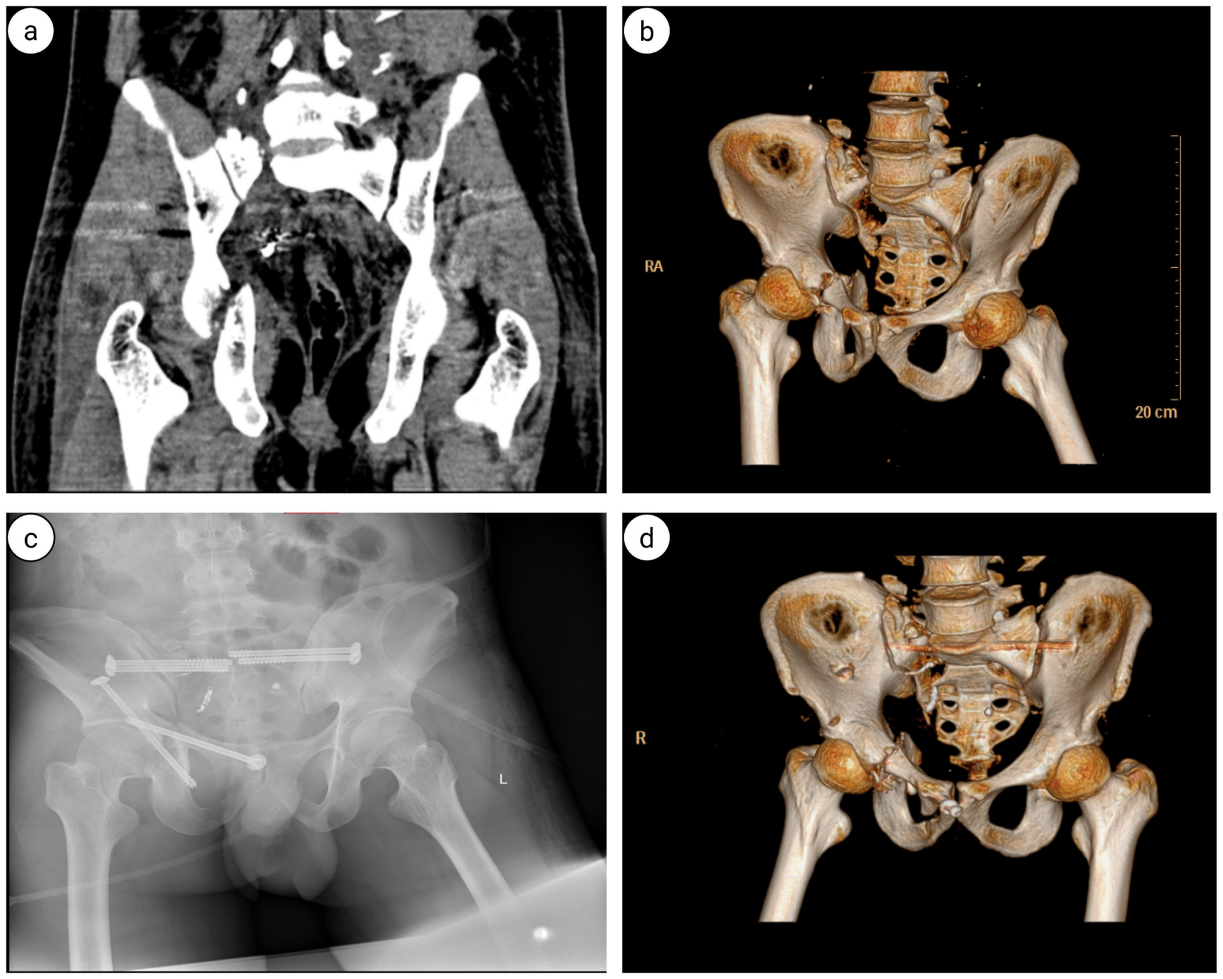
Preoperative and postoperative digital radiography (DR) and three-dimensional computed tomography (3D CT) of pelvic and acetabular fracture: **(a)** Preoperative pelvic anteroposterior X-ray; **(b)** preoperative pelvic computed tomography (CT) three-dimensional reconstruction; **(c)** postoperative day one pelvic anteroposterior X-ray; **(d)** postoperative day one pelvic CT three-dimensional reconstruction.

The Pelvic Unloading and Reduction Device (UCRT), patented under number CN201510543929.3 and developed by Shijiazhuang High-tech Zone Yicheng Technology Co, Ltd., represents a significant advancement in the field of orthopedic surgical devices. This specialized medical apparatus is engineered to facilitate the reduction and stabilization of unstable posterior pelvic ring injuries, thereby enhancing surgical precision and patient outcomes. The UCRT is designed to operate in conjunction with a comprehensive fluoroscopy operating table equipped with rigid connection traction, lead shielding, and advanced fluoroscopy imaging systems. Notably, the rigid connection traction component can be substituted with a modular setup comprising a traction table used in intertrochanteric femoral fracture surgeries and a wooden bed, offering flexibility tailored to various surgical environments. Traction is applied through nylon ropes connected to a traction bow, exerting longitudinal force on the patient’s limb to assist in the reduction of severely displaced pelvic fractures (30). The device’s architecture aims to improve reduction accuracy and stability during operative procedures. Complementing the UCRT is the Holosight Robot, developed by researchers including Chen Hua and Cao Wenhao. This robotic system employs voxel registration technology to achieve high-precision surgical outcomes. Fully independent in terms of intellectual property rights, the Holosight Robot has been widely adopted for the intelligent, monitored reduction of unstable pelvic fractures and sacroiliac screw placement. Its application has demonstrated notable benefits, including enhanced reduction quality, reduced radiation exposure to medical personnel, and increased accuracy in screw placement (27, 31–33). When integrated with the UCRT, the Holosight Robot enables real-time three-dimensional visualization of bone morphology and positioning. This integration facilitates highly accurate reduction and fixation through computer-aided automatic planning and high-precision optical tracking technology, representing a significant advancement in minimally invasive pelvic fracture management (34). Preoperative assessment involves detailed analysis of pelvic and acetabular fractures utilizing X-ray imaging and three-dimensional computed tomography (CT). Based on this comprehensive evaluation, the surgical team formulates a reduction plan, often selecting the uninjured side as a reference point. In a typical case, a type C fracture of the right pelvis with a displaced transverse acetabular fracture is identified, with the contralateral side remaining intact. During surgery, the Holosight Robot guides the procedure by inserting Kirschner wires into strategic anatomical landmarks, such as the junction of the iliac tuberosity at the superior edge of the left acetabulum and the right pelvis. These wires are secured to the UCRT, creating a stable reduction unit. Traction is applied laterally and longitudinally, with real-time monitoring via the robot’s display to correct vertical displacement. Rotational displacements are managed through controlled manipulation of the Kirschner wires, with continuous visualization ensuring precise correction of internal, external, superior, and inferior rotations. Following confirmation of satisfactory reduction through fluoroscopy in multiple views, the surgical team proceeds with screw placement. Using the three-dimensional planning capabilities of the Holosight Robot, entry points for sacroiliac screws (S1 and S2), pubic ramus screws, and posterior column screws are meticulously determined. Guide wires are inserted accordingly, with their trajectories verified fluoroscopically (Figures 15, 16). Subsequently, cannulated screws are implanted along these guide wires to secure the pelvis and acetabulum. Final fluoroscopic imaging confirms optimal screw positioning and fracture reduction (Figure 17). The procedure concludes with the removal of the reduction frame, irrigation, and suturing of small incisions, minimizing tissue trauma and promoting recovery. This integrated approach exemplifies the latest advancements in minimally invasive pelvic fracture treatment, emphasizing precision, safety, and improved patient outcomes.

## 5 Discussion

### 5.1 The importance of early evaluation-diagnosis-surgical repair for traumatic diaphragmatic injury (TDI) in polytrauma

Polytrauma describes an injury pattern where a single mechanical trauma affects multiple anatomical sites either simultaneously or sequentially. The ISS classifies polytrauma into four categories: mild (ISS < 9), moderate (9 ≤ ISS < 16), severe (16 ≤ ISS < 25), and critical (ISS ≥ 25) (5). Managing polytrauma remains a significant clinical challenge, characterized by four distinctive features: high incidence of shock, hypoxemia, mortality, and infectious complications (35). When treating critical polytrauma cases complicated by traumatic diaphragmatic hernia and pelvic and acetabular fractures—particularly in high-energy trauma—the diagnostic approach should extend beyond the pelvic region. A thorough systematic physical examination, along with timely imaging and hematological studies, is essential to identify potential TDI, associated organ damage, and sources of hypovolemic shock (36). While diaphragmatic injury is relatively rare in trauma patients, its diagnosis holds substantial clinical significance (37). Research demonstrates that high-energy trauma frequently results in concurrent diaphragmatic rupture, thoracic vertebral injuries, and pelvic injuries, correlating with ISS scores and ICU duration (38). Acute diaphragmatic hernia develops following diaphragmatic injury associated with severe blunt or penetrating thoracoabdominal trauma, with TDI categorized as either blunt or penetrating (39, 40). TDI represents an uncommonly diagnosed condition that may present without obvious clinical manifestations. Traffic accidents constitute the primary cause, and concurrent thoracic and abdominal injuries often obscure its presence (41). Untreated TDI complications, particularly diaphragmatic hernia with organ strangulation, can precipitate rapid clinical deterioration. Specifically, diaphragmatic hernia with displaced organ involvement may induce acute respiratory failure and cardiac arrest, posing immediate life-threatening consequences. Hanna et al. reported that 43.8% of TDI patients develop acute diaphragmatic hernia, predominantly involving abdominal organ herniation. The stomach represents the most frequently herniated organ, followed by the colon, spleen, omentum, kidney, and small intestine (42, 43). Common musculoskeletal injuries associated with TDI include fractures of the ribs, pelvis, spine, skull, and long bones (44). In this case, high-energy trauma induced diaphragmatic injury resulting in acute left diaphragmatic hernia. The herniation of stomach, colon, omentum, and spleen into the thoracic cavity led to lung compression, precipitating two episodes of acute respiratory failure. Diaphragmatic rupture from blunt chest trauma constitutes a severe acute condition requiring surgical intervention. Therefore, prompt evaluation, diagnosis, and surgical repair of traumatic diaphragmatic injury prove crucial for patient survival and facilitate optimal conditions for subsequent fracture surgery (45).

### 5.2 Comparison between orthopedic surgical robot-assisted minimally invasive surgery for pelvic and acetabular fractures and traditional ORIF

Pelvic and acetabular fractures present significant challenges in traumatic orthopedic treatment. These fractures constitute 3% of all fractures, typically resulting from high-energy injuries, and are associated with considerable mortality and disability rates. The primary objective of surgical intervention for pelvic and acetabular fractures is to restore anatomical symmetry and stability of the pelvic ring and acetabulum (46). ORIF represents the gold standard treatment approach. However, traditional ORIF presents several limitations, including extensive trauma, substantial intraoperative blood loss, risks of vascular or neural injury, elevated infection rates, heterotopic ossification, femoral head avascular necrosis, and delayed rehabilitation. Despite continuous refinements in traditional surgical techniques to minimize invasiveness, these approaches cannot match the effectiveness of robot-assisted minimally invasive surgery. The rapid evolution of robotics has facilitated its integration into clinical practice, offering new possibilities for minimally invasive, precise, and personalized treatment. The introduction of RoboDoc in 1991—the first orthopedic surgical robot—marked a significant milestone, with its clinical trial completed that same year. Recent advances in surgical robotics have expanded their application in orthopedics. In 2015, Beijing Jishuitan Hospital developed the “Tianji” Robot System, pioneering intelligent orthopedic surgical treatment and introducing robotic assistance for percutaneous minimally invasive acetabular fracture fixation (47). This robotic system enables surgeons to place screws with enhanced safety, accuracy, and stability during minimally invasive internal fixation procedures. Orthopedic surgical robots have demonstrated superior clinical outcomes compared to traditional ORIF in treating pelvic and acetabular fractures. While traditional fluoroscopic-guided screw placement risks malposition, robot-assisted surgery ensures precise screw placement through preoperative imaging, real-time tracking, and robotic arm guidance (48). Multiple studies validate these advantages: Zhao et al. (49) documented superior outcomes in 18 acetabular fracture cases using HoloSight robot-assisted two-window screw implantation, reporting reduced screw placement time, fewer fluoroscopies, and improved implantation quality with a 90% excellent reduction rate. Cao et al. (50) demonstrated enhanced outcomes using the Unlocked Closed Reduction Tool (UCRT) with HoloSight in 13 Tile Type C1 pelvic fractures, achieving shorter reduction times and 100% screw placement accuracy. Zhao et al. (51) reported a 95.5% excellent reduction rate using robot-assisted minimally invasive pelvic reduction in 22 unstable pelvic fractures. Xue et al. (52) achieved 100% accuracy in subacetabular screw placement using robotic navigation. Robot-assisted minimally invasive treatment offers comprehensive advantages: preoperative path planning, precise intraoperative guidance, minimal trauma, reduced complications, preserved femoral head blood supply, and enhanced rehabilitation outcomes. The clinical benefits include aesthetic incisions, expedited rehabilitation, shortened treatment cycles, reduced operation time, decreased radiation exposure, and lower complication rates.

### 5.3 Trauma care process: key pathway for enhancing rescue efficacy in critical polytrauma patients

**Figure 18:**
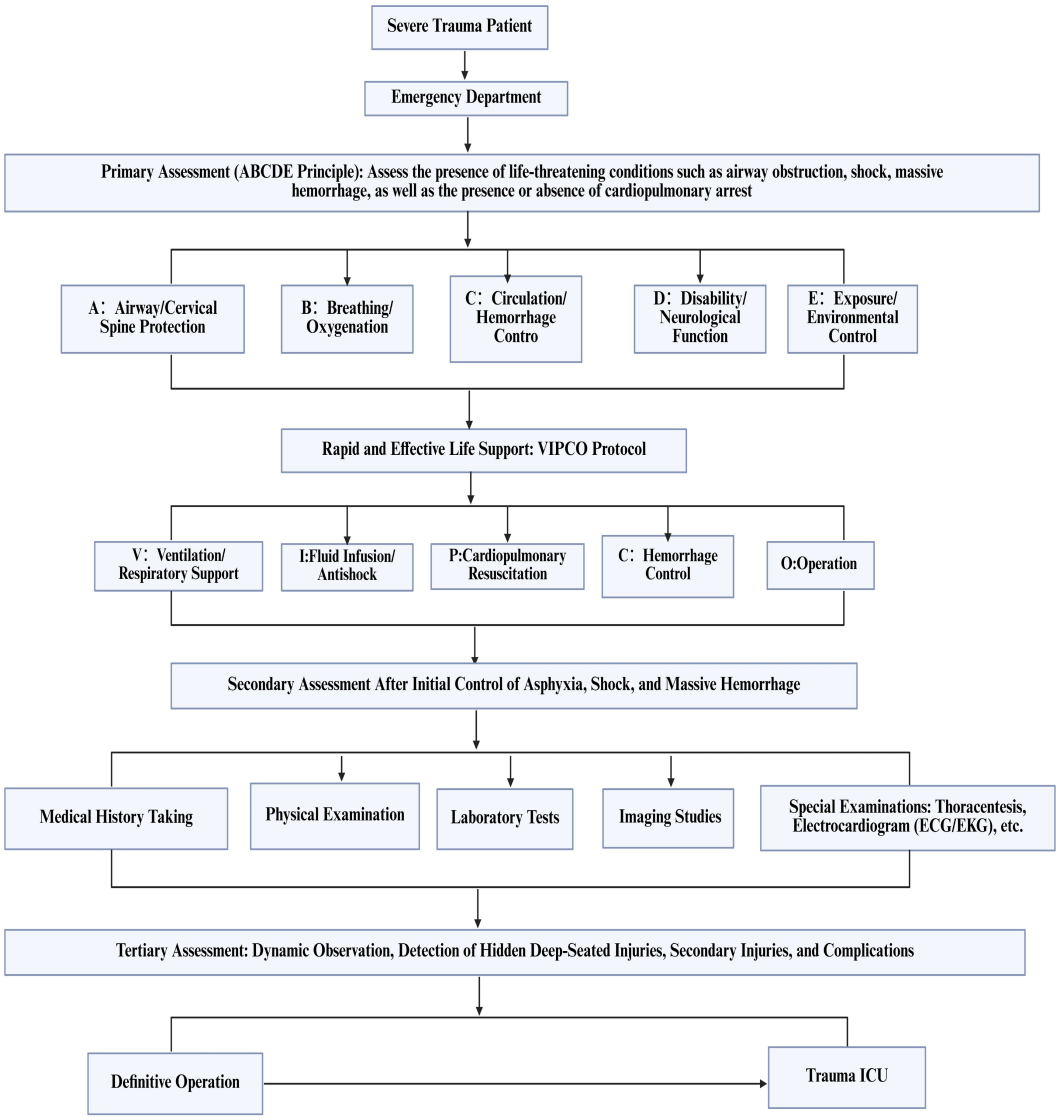
In-hospital treatment flow chart for severe trauma patients.

**Figure 19:**
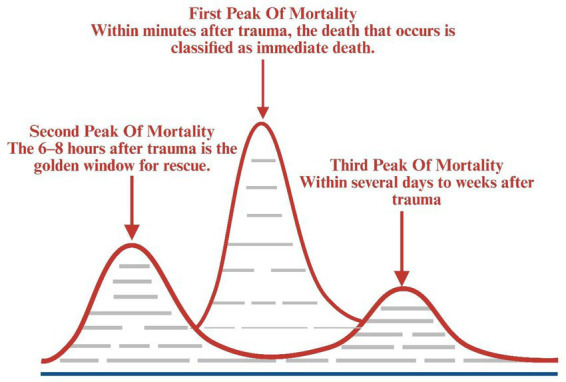
Three major peak periods of death in polytrauma.

Orthopedic surgical robots have shown increasing maturity in the diagnosis and treatment of pelvic and acetabular fractures. However, postoperative complications and surgical failures still occur. Therefore, successful treatment requires both a solid foundation in basic knowledge and traditional methods, as well as comprehensive initial patient assessment. In treating critical polytrauma patients, scientific evaluation of the trauma care process remains the core determinant of treatment success, maintaining its significance throughout the entire treatment course. Early trauma patient assessment comprises primary assessment (ABCDE Principle), secondary assessment (CRUSHPLAN Principle), and tertiary assessment. Given that trauma is highly time-dependent, effective assessment and management must occur within minimal timeframes. Dynamic assessment should follow the principles of “assess first, then diagnose” and “assess while rescuing”. In this patient’s treatment, our hospital strictly adhered to the In-Hospital Trauma Care Process for Severe Trauma Patients (Figure 18), providing high-quality and timely assessment, resuscitation, and treatment during the second and third peak periods of trauma-related death (Figure 19). The three major peak periods of trauma-related death, first proposed by Trunkey (53), include: death within minutes to 1 h after trauma (first peak); death within 6–8 h after trauma (second peak); and death within days or weeks after trauma (third peak). These peaks represent approximately 50, 30, and 20% of total trauma-related deaths, respectively. The first hour post-trauma is termed the “Golden Hour”. Implementation of rapid and effective assessment and resuscitation measures during the early peak period can reduce preventable deaths from 35% to less than 10% (54). Comprehensive trauma patient assessment facilitates early identification of optimal surgical timing and planning for combined pelvic and acetabular fractures, thereby improving survival and cure rates while reducing disability and mortality rates.

### 5.4 Postoperative management and early rehabilitation exercises

Following minimally invasive treatment for pelvic and acetabular fractures, patients receive symptomatic therapies such as edema reduction, analgesia, and infection prevention. On the first postoperative day, low-molecular-weight heparin is administered to prevent deep vein thrombosis, accompanied by ankle pump exercises. Patients are encouraged to perform turning activities in bed and engage in isometric and isotonic muscle contractions of the lower limbs. On the second postoperative day, active range-of-motion exercises for the hip and knee joints are initiated to prevent joint stiffness and muscle atrophy. Four weeks after surgery, on May 30, 2023, patients return for follow-up appointments and begin partial weight-bearing gait training using crutches. Full weight-bearing ambulation is commenced at 8 weeks post-surgery, on June 30, 2023.

### 5.5 “Minimally invasive fracture technology + optimization of trauma care process + seamless collaboration of multidisciplinary team” jointly achieve rapid postoperative recovery in critical polytrauma patients (ERAS concept)

Patient postoperative rehabilitation significantly correlates with injury severity. Rapid recovery of joint function represents a fundamental aspect of post-surgical care for pelvic and acetabular fractures (55). Early postoperative mobilization represents a crucial measure for rapid rehabilitation and shortened hospital stays, aligning with ERAS core principles (56). ERAS optimizes surgical patient care across preoperative, intraoperative, and postoperative phases based on evidence-based medicine. This approach reduces postoperative complications, shortens hospital stays, improves postoperative quality of life, and decreases overall medical costs. Danish surgical professor Henrik Kehlet first introduced Fast Track Surgery (FTS) in 1997. Following continuous development, this model gained clinical surgical implementation. Academician Li Jieshou introduced fast-track rehabilitation to China in 2006, leading to its gradual adoption across multiple surgical disciplines. ERAS guidelines are multimodal, involving multidisciplinary teams of surgeons, anesthesiologists, nurses, and allied health professionals. Early mobilization, a crucial ERAS component, reduces surgical stress load and adverse physiological reactions from fixation procedures, decreases postoperative complication risks, accelerates functional walking recovery, positively impacts patient-reported outcomes, and reduces hospital stays and nursing costs (57). Throughout this patient’s treatment, our hospital’s trauma care process strictly followed ERAS principles with patient-centered care. Through seamless multidisciplinary collaboration—including emergency department dynamic management, ICU monitoring, anesthesiology preoperative assessment, combined gastrointestinal and thoracic surgery, trauma orthopedics minimally invasive treatment, and rehabilitation department early functional exercises—we optimized the trauma care process across all phases. This approach reduced postoperative traumatic stress response, enabled early functional exercises, promoted rapid rehabilitation, and achieved precise, minimally invasive treatment. ERAS success depends not only on multidisciplinary collaboration and high-standard, evidence-based guidelines (58) but also on patient participation and medical team guidance throughout preoperative preparation, intraoperative cooperation, and postoperative rehabilitation.

Nevertheless, this report exhibits several potential limitations. First, it includes only one patient, which precludes the ability to draw definitive conclusions. Second, the minimally invasive techniques employed for treating pelvic-acetabular fractures depend on advanced equipment and must be performed by specialized teams, which precludes their widespread adoption as a standardized treatment protocol. Finally, efficient collaboration within the Multidisciplinary Team (MDT) and the Enhanced Recovery After Surgery (ERAS) management relies on cooperation among various departments, including the Emergency Department, Thoracic Surgery Department, Anesthesiology Department, Intensive Care Unit (ICU), and Orthopedics Department. This model presents challenges for other medical institutions to fully replicate, thereby limiting the protocol’s reproducibility. In 2024, the team led by Academician Tang Peifu and Professor Chen Hua developed a remote trauma orthopedic surgical system utilizing the HoloSight trauma orthopedic surgical robot combined with the Pelvic Unlock and Reduction Device (UCRT). This system enables expert remote guidance during surgical procedures, real-time monitoring of reduction outcomes, planning of screw trajectories, and selection of appropriate screws, thereby facilitating interactive remote surgeries. The technology successfully treated a patient with pelvic ring injury, specifically a Tile C1.3 type pelvic ring fracture (59). Future research should focus on larger sample sizes and prospective, multicenter, randomized controlled trials with long-term follow-up to validate the advantages of this technology in remote treatment of pelvic ring fractures and to comprehensively assess its clinical efficacy in patients with severe multiple trauma associated with traumatic diaphragmatic hernia (TDH), pelvic fractures, and acetabular fractures.

## 6 Conclusion

In summary, this report provides comprehensive clinical data and therapeutic strategies for the diagnosis and management of critical polytrauma complicated by traumatic diaphragmatic hernia (TDH), pelvic fractures, and acetabular fractures. Furthermore, the report underscores the significance of optimizing trauma care workflows and fostering collaboration within multidisciplinary teams (MDT). By prioritizing the management of life-threatening injuries associated with TDH and employing minimally invasive treatments facilitated by orthopedic surgical robots, the team collectively achieved expedited postoperative recovery.

## Data Availability

The original contributions presented in the study are included in the article/Supplementary material, further inquiries can be directed to the corresponding author.
