# Correction: Minimally invasive treatment for critical multiple trauma with traumatic diaphragmatic hernia combined with pelvic and acetabular fractures: a rare case report

**DOI:** 10.3389/fmed.2026.1856627

**Published:** 2026-05-08

**Authors:** Meng-Fei Zhou, Zeng-Ying Xing, Ri-Jin Xing, Tian-Yang Li, Bi-Gang Li, Wei Liu, Yun-Yun Wu, Jun Xiong

**Affiliations:** Hainan General Hospital, Hainan Affiliated Hospital of Hainan Medical University, Haikou, China

**Keywords:** combined pelvic and acetabular fractures, HoloSight Orthopedic Surgical Robot, multidisciplinary joint surgery, severe polytrauma, traumatic diaphragmatic hernia

Author ‘Jun Xiong' was erroneously assigned as corresponding author. The correct co- corresponding authors are ‘Jun Xiong and Yun-Yun Wu'.

The original version of this article has been updated.

